# Distinct Spectral Profiles of Pleural Effusions from Malignant Tumors Using Raman Spectroscopy

**DOI:** 10.3390/ijms262311707

**Published:** 2025-12-03

**Authors:** Monika Kujdowicz, Piotr Jeleń, Maciej Sitarz, Marta Marcinek, Janusz Włodarczyk, Michał Wiłkojć, Lucyna Rudnicka, Dariusz Adamek

**Affiliations:** 1 Department of Pathomorphology, Jagiellonian University Medical College, Grzegórzecka 16, 31-531 Krakow, Poland; 2 Department of Pathomorphology, University Children’s Hospital of Krakow, Wielicka 265, 30-663 Krakow, Poland; 3 Faculty of Materials Science and Ceramics, AGH University of Krakow, Al. Mickiewicza 30, 30-059 Krakow, Poland; pjelen@agh.edu.pl (P.J.); msitarz@agh.edu.pl (M.S.); 4 Department of General Thoratic Surgery, John Paul II Hospital, 31-202 Krakow, Poland; 5 Department of General, Oncological, Metabolic and Thoracic Surgery, Military Institute of Medicine, National Research Institute, 04-141 Warsaw, Poland; 6 Department of Pathology, John Paul II Hospital, 31-202 Krakow, Poland

**Keywords:** raman spectroscopy, mesothelioma, lung carcinoma, metastases to lung, pleural effusion

## Abstract

Raman spectroscopy is a powerful method in the field of cancer diagnosis, for which various sample types and measurement modalities can be used. In this study, pleural effusion samples from twenty patients with suspected malignancies were analyzed. After fluid samples were fixed with ethanol and dried, high-quality spectra were taken at three different points using two laser lines. Principal Component Analysis showed clustering of spectra from malignant samples. The results show that despite a strong fluorescence signal from blood with the 532 nm laser line, spectra collected with both 532 nm and 785 nm laser lines are complementary, as they produce different high-intensity bands; e.g., breast cancer and adenocarcinoma signals are stronger with 785 nm. The main change in cancer specimens is an increase in amino acids. In addition, in small-cell carcinoma of the lung and mesothelioma, elevated nucleic acids and lipids were observed, respectively. Raman spectroscopy shows distinct profiles for control and malignant effusions. Further investigation of the utility of spectral markers in personalized treatment could improve survival.

## 1. Introduction

Pleural effusion is quite a rare condition, but it occurs in cases of extensive, life-threatening lung inflammation, congestive heart failure, liver cirrhosis, pulmonary embolism, and tumors (10%). It can occur with kidney and autoimmune diseases, though this is rare. Effusion can result from increased fluid production when neoplastic cells infiltrate capillaries, and tumor-related inflammation with cytokine production increases their permeability. It can also occur when lymphatic cells are blocked by tumor cells, which decreases lymphatic drainage and further impairs fluid reabsorption. More fluid is present in malignant effusions than in non-malignant cases [1]. The most common malignancies producing pleural effusion are lung (squamous carcinoma and adenocarcinoma) and breast cancer, as well as metastases from the colon and female genital organs. Less frequently, pleural effusion occurs in mesothelioma and lymphomas [2,3]. Lung and breast cancer rank high in malignancy incidence and mortality rate worldwide [4]. Mesothelioma, although rare, primarily grows in the pleural cavity; its symptoms include chest pain and breathlessness, which are largely (in 70%) attributable to effusion [5]. Metastasis, diffuse chest wall or mediastinal involvement, and transmural infiltration are often observed in the advanced stage of mesothelioma. Pleural effusion is present in advanced stages of breast and colon metastases, various stages of lung cancer, and in the early stage of mesothelioma [6]. Blood test markers have been identified for many cancers but are yet to be established for lung cancer and mesothelioma, which are diagnosed through radiology of the chest and invasive tissue biopsies.

Effusion cytology is a rapid, highly specific but low-sensitivity test for neoplasms. Metastases are diagnosed on the basis of medical history and the presence of multiple, various-sized tumors. In cases of poorly differentiated cancers and multiorgan disease, the primary site is hard to specify. In all types of secondary-site cancer, spread to the lungs significantly increases the clinical stage to stage M (metastases), meaning distant spread, with a very poor prognosis. The choice of therapy depends on the tumor’s origin, which in turn involves numerous radiological examinations and biopsies for histology, along with various immunohistochemical tests. Therefore, “rapid diagnostic tools” such as pleural fluid spectroscopy can be vital in accelerating therapeutic decisions and initiating targeted treatment.

A novel alternative is based on biochemical “fingerprint” analyses. The principle of vibrational spectroscopy is the detection of chemical bond vibrations according to their energy (wavelength/wavenumber of electromagnetic wave) [7]. Infrared and Raman (RS) spectroscopy are both semiquantitative, complementary methods that are sensitive to various biochemical components according to vibration symmetry. RS uses lasers to obtain non-elastic scattering spectra, presenting multiple bands for the biological sample. Raman spectra vary in band intensities, spectral shifts, and fluorescence depending on the laser line used [8]. RS has been successfully used to classify many malignancies [9,10,11].

The aim of this study was to provide a proof of concept for the use of RS of pleural effusions for cancer diagnosis, as well as to select the best laser line and the most useful bands and spectral range.

## 2. Results

### 2.1. Patients’ Standard Cytology

The subjects were 9 females and 11 males, with an average age of 71 years (range 57–88). The control group, with no cancer in cytology or medical history, comprised five cases of reactive changes. The malignancy group included four mesotheliomas (Mes), three adenocarcinomas (AdCa) of the lung and one of the colon, two squamous lung carcinomas (SqCa), two ovarian serous carcinomas (OvCa), one case of small-cell lung carcinoma (SCC), one breast carcinoma (poorly differentiated), and one renal cell carcinoma (RCC, clear cell subtype). All samples contained vivid neoplastic cells in standard cytology. Detailed morphological information from cytology is presented in Table 1.

### 2.2. Raman Spectra and Biochemical Components of Samples

**Figure 1:**
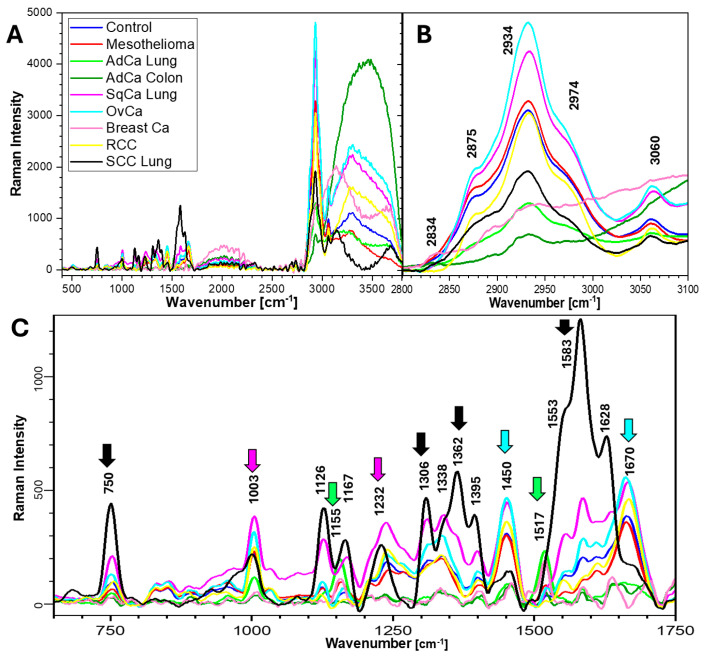
Averaged Raman spectra of patient effusions, 532 nm laser line. The arrows mark the most characteristic bands for the groups (according to colors). (**A**) 400–3550 cm^−1^ region; (**B**) high-number region; (**C**) fingerprint region.

**Figure 2:**
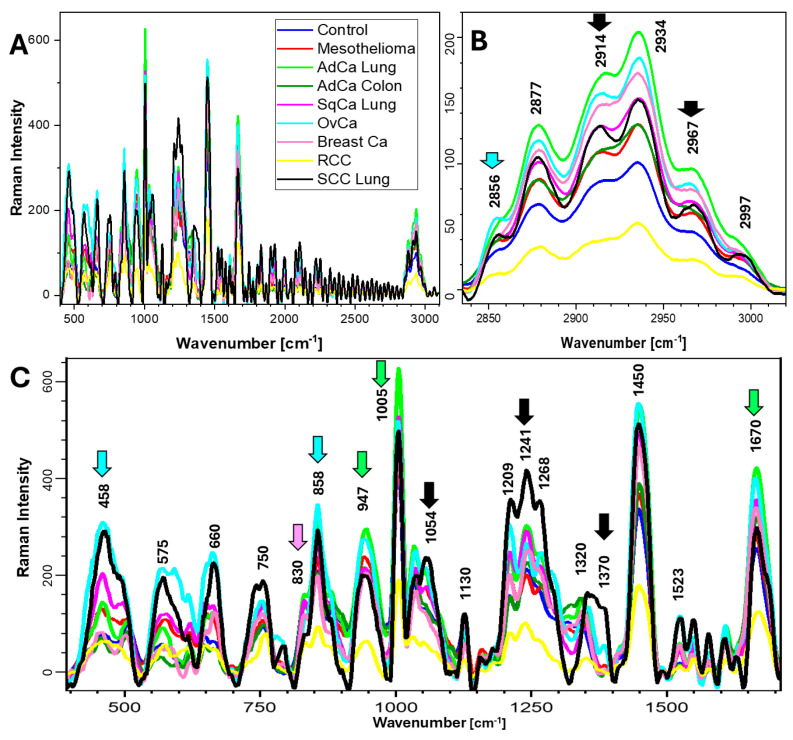
Averaged Raman spectra of patient effusions, 785 nm laser line. The arrows mark the most characteristic bands for the groups (according to colors). (**A**) 400–3100 cm^−1^ region; (**B**) high-number region; (**C**) fingerprint region.

Both laser lines (532 and 785 nm) produced good signals in the fingerprint range (400–1800 cm^−1^) and high-number bands (2800–3500 cm^−1^) in the control sample. Blood fluorescence signals (approximately 2000 cm^−1^ and 3500 cm^−1^) were present with the 532 nm line (Figure 1). The intensities of high-number bands were higher with the 532 nm line, while those of the fingerprint bands were higher with the 785 nm line (Figure 2).

RS at 532 nm showed that SCC had the highest band intensities at 1583 cm^−1^ (proteins and nucleic acids; stretching ν(C=C)), 750 cm^−1^ (nucleic acids, tryptophan [Trp], and cytochromes; CH_2_ rocking, symmetric breathing), and 1362 cm^−1^ (nucleic acids and proteins)—see Table 2. Further, SCC also had high intensities at 1553 (amide II, (amide I and cytidine in nucleic acids; ν (NH)) and 1628 cm^−1^ (amide I β-sheet configuration and Trp, Tyr; ν(C=O)). SqCa spectra had the highest intensities at 1003 cm^−1^ (phenylalanine residues), 1232 cm^−1^ (amide III, Trp; stretching ν(C-N); and in-plane deformations δ(N-H)), and 1338 cm^−1^ (proteins and carbohydrates; δ(CH)). Both SCC and SqCa had high Trp (1126 cm^−1^; *v*(CN) and *v*(CC)) and amide III (1395 cm^−1^; *v*(CN), *v*(CC), and *v*(CH) of amino acids). Mesothelioma had a low intensity signal in the fingerprint region, beside medium intensity of 1450 cm^−1^ (...) and 1670 cm^−1^ (...), but it had high-intensity bands in the 285–3000 cm^−1^ region. With the 532 nm laser line, the high-number region showed high levels of bioorganic matter and fat in OvCa and AdCa of the colon (2875 cm^−1^—ν(C-H)-CH_2_; 2934 cm^−1^—ν(C-H); 2974 cm^−1^ [this band is also assigned to nucleic acids]—ν_as_(CH_3_), ν_as_CH(-CH_2_); and 3060 cm^−1^—ν(=C-H)). Interestingly, AdCa of the lung and breast Ca produced high-intensity signals in the high-number region with 785 nm, while they were low with 532 nm, though the reason for this phenomenon is unclear. The concentration does not change the spectral profiles, and the measurements were performed on dried pellets of the effusions (powder volume was c.a. 3 × 3 × 1 mm). Pellets were dried for 24 h at 40 °C, additionally dried with the laser beam until signal stabilization, and then measured. The measurements were performed with 532 nm and 785 nm lasers one by one, sample after sample. We did not observe burning while irradiating the samples. We suspect that the heme group, which is seen at 532 nm, enhanced the low bioorganic signal at 785 nm, and this phenomenon is especially pronounced in the high-number region. The 830 cm^−1^ band is assigned to Tyr interactions of proteins (assigned to the Tyr ring-breathing mode, which is sensitive to the amino acid’s environment and conformation) [12,13]. The presence of the 830 and 856 cm^−1^ doublet is used to investigate protein conformation; this analysis is based on the Fermi resonance effect between the phenol ring’s planar and nonplanar modes [14]. In a subset of mesothelioma spectra with lower intensity in the high-number region with 532 nm, we observed that their intensity in this region inverted, becoming higher, which was also related to the presence of 856 cm^−1^. AdCa had high intensity at 1155 cm^−1^ (tyrosine [Tyr] and phenylalanine [Phe]) and a specific signal at 1517 cm^−1^ (Tyr). For RCC, intensities were quite high at 1670 cm^−1^ and 1450 cm^−1^ but low at other bands. Breast carcinoma bands were low or almost absent for Tyr in 532 nm laser measurements (possibly due to low cancer cell number), but they could be detected by the 785 nm laser line, where the amide III region (1200–1400 cm^−1^) and high-number region had high-intensity bands.

### 2.3. Principal Component Analysis (PCA)

**Figure 3:**
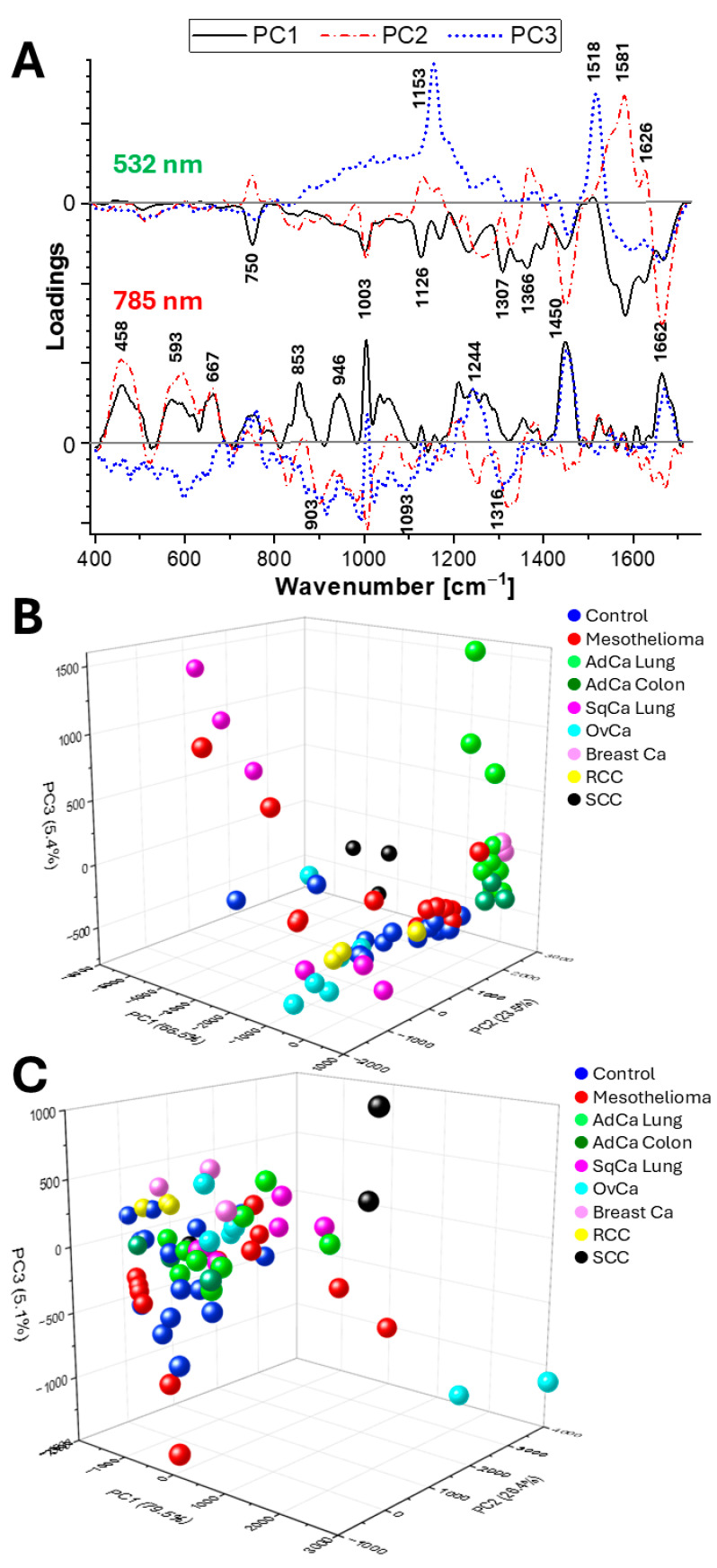
PCA of three spectra from each patient’s effusion. (**A**)—Loadings; (**B**,**C**)—scores for 532 nm and 785 nm laser lines, respectively.

Principal Component Analysis (PCA) is a fast, straightforward chemometric method for explaining the majority of variability in the data, which does not require knowledge of the biochemical compounds in the samples [17]. For both laser lines, the analysis of three spectra from each of the 20 patients showed the best clustering of experimental groups for the fingerprint region (400–1720 cm^−1^), as presented in Figure 3. We also tested the high-number region separately (2800–3050 cm^−1^) and both regions together, with slightly less satisfactory outcomes.

PCA of spectra collected with a 532 nm laser line shows variances of 62.5%, 22.5%, and 5.4% for PC1, PC2, and PC3, respectively. The first principal component differentiates SqCa and SCC from the other groups. Changes in PC1 involved negative vectors for 1581, 1366, 1307, 1126, 1003, and 750 cm^−1^ (mainly assigned to proteins, nucleic acids, Trp, Tyr, Phe, and phospholipids). PC2 was high for adenocarcinomas and breast cancer, medium for control and mesothelioma, and low for RCC, SqCa, and OvCa. Changes in PC2 involved positive vectors for 1626, 1581, and 750 cm^−1^ (proteins) and negative vectors for 1662 and 1450 cm^−1^ (lipids, Trp, and proteins). PC3 was higher for mesothelioma than for the control group. Changes in PC3 involved positive vectors for 1518 and 1153 cm^−1^, assigned to Tyr.

PCA of spectra collected with a 785 nm laser line shows variances of 79.5%, 26.4%, and 5.1% for PC1, PC2, and PC3, respectively. PC1 differentiates OvCa, SCC, and adenocarcinomas from the other groups. Changes in PC1 involved positive vectors for 1662, 1450, 1244, 1003, 946, 853, and 667 cm^−1^ (assigned to proteins Phe, Tyr, polysaccharides, Tyr, and lipids [carbonyl ν(C=O)]). PC2 was low for mesothelioma and high for OvCa, SCC, and SqCa. Changes in PC2 involved negative vectors for 1316, 1003, and 903 cm^−1^ (Tyr and Phe) and positive vectors for 667, 593, and 458 cm^−1^ (assigned to lipids and nucleic acids). PC3 was higher for breast carcinoma, RCC, and OvCa and low for mesothelioma. PC3 has positive vectors for 1662, 1450, and 1244 cm^−1^ (proteins) and negative vectors for 1316, 1093, and 903 cm^−1^ (Trp, nucleic acids, lipids, and hydroxyproline).

## 3. Discussion

### 3.1. RS of Pleural Effusions

The literature on Raman spectroscopy of pleural effusion applied in cancer diagnosis is scarce. We found two works with the usage of pleural effusion but with different methodology than ours (preservation of samples (frozen and stored at −82 °C, and with additional enhancement of RS signal generated by silver nanoparticles and analyzed spectral region from 600 cm^−1^ to 1800 cm^−1^). 

Adenocarcinoma, among other groups, had the highest 1133 cm^−1^ band intensity (lipids and proteins). Our study shows similar biochemical differences between squamous carcinoma and controls, but in cases of adenocarcinoma, we found low protein levels. One possible cause of the different protein levels might be that our lung and colon AdCa samples contained different amounts of live cancer cells and necrotic changes; indeed, high levels of the amino acid residues Phe and Tyr were preserved. Protein fragmentation decreases the band intensity, as demonstrated in cases of cancer infiltration [18]. Kowalska et al. analyzed 14 malignant effusions (adenocarcinoma and squamous cell carcinoma) and 6 controls with a 785 nm laser line using surface-enhanced Raman scattering (SERS) spectroscopy [19]. They collected maps of the fluid sediment. The spectra presented by this group were shifted compared to ours due to the different methodology used. They found that the groups differed in intensity at multiple bands. For both malignancies, intensity was higher at 1003 cm^−1^, 1206 cm^−1^, and 1445 cm^−1^ (proteins, especially collagen in tissues) and lower at 1610 cm^−1^ (Phe, Tyr, and cytosine) compared to controls. Squamous carcinoma had the highest intensities at 633 cm^−1^ (Phe), 890 cm^−1^, and 1397 cm^−1^ (proteins).

We found higher intensities at 637 cm^−1^ (Tyr), 1131 cm^−1^ (lipids and proteins), 1217 cm^−1^ and 1668 cm^−1^ (proteins, amide III and amide I, respectively), and 1580 cm^−1^ (proteins and DNA) and lower intensities at 1325 cm^−1^ (lipids) and 1445 cm^−1^ (proteins) in malignancies compared to the benign group. Liu et al. reported similar malignancy features, besides lower nucleic acid content. They used an OPLS-DA model for SERS spectra classification (leave-one-out cross-validation; 15 spectra/patient) and a 785 nm laser line, and the samples originated from 32 controls and 51 malignant pleural effusion patients (tumor types not listed) [20]. Sensitivity and specificity were c.a. 92% and 94%, respectively.

Taken together, the most common characteristic of cancer is an increased level of amino acid residues, while protein and lipid band intensities can vary depending on chemical group vibrations. This work demonstrates that the two tested laser lines are complementary, as some differences are more pronounced in one or the other (e.g., the low signal for breast cancer and adenocarcinoma at 532 nm is clearly detectable at 785 nm; similarly, SCC is characterized by very high 1583 cm^−1^ intensity at 532 nm, while it is lower at 785 nm).

### 3.2. The Value of Pleural Samples

Pleural fluid is a valuable sample type that can be used for cell culture as part of personalized therapy. Cell cultures are often used in basic sciences (methodology, pharmacy), while patient cohort samples are used for clinical diagnosis and treatment efficiency, and the required sample size depends on disease occurrence. Tissues are more complex; they contain a microenvironment consisting of the extracellular matrix, non-cancer cells (e.g., cancer-associated fibroblasts and endothelial and immune cells), and extracellular fluid. The difference between tissues and cell cultures affects clinical translation—can cell cultures reflect the changes in the human body?

Lung cancer and mesothelioma are among the most lethal cancers, and there are hundreds of commercially available cell lines; for example, there are approximately 136 established cell lines for mesothelioma [21], which were derived from epithelioid, sarcomatoid, desmoplastic, and biphasic subtypes, all with multiple gene alterations. The sarcomatoid subtype has worse outcomes; the cells are longer, and they undergo epithelioid-to-mesenchymal transition. This transition, also called dedifferentiation (broader definition), is present in many tumors. In vivo models, such as asbestosis-induced and conditional mouse models, produce results more consistent with clinical findings; however, while these models can be used to investigate general treatment for different cancer subtypes, personalized treatment is the best choice for multidrug-resistant tumors. Established cell line cultures might not contain certain metabolites, such as carbohydrates, and further, different preservation methods have varying effects on the glycogen bands [9,18]. Zipprick et al.’s review showed a positive correlation between the drug sensitivity of patient-derived cells ex vivo and the clinical outcomes for personalized treatments of non-small-cell lung cancer and mesothelioma [22]. With patient-derived culturing, they concluded that the pleural effusion cells and their 3D cultures present advantages over other approaches in ex vivo tests.

Besides the sample type, certain methods can enhance the signal, such as surface-enhanced Raman spectroscopy (SERS) [23]. The literature broadly describes the methodology and application of RS in biomedicine; e.g., in oncology, infectious disease, and treatment monitoring [24]. SERS platforms enhance miniaturization capability and measurement sensitivity and can be used at the point of care, outside of specialized labs [25]. The technique was proven to detect early-stage lung cancer from serum samples [26].

### 3.3. RS of Tumors Detected in Pleural Effusions

A literature search returns many RS articles on lung cancer, with sample types including blood, tissue, and saliva [27,28]. Previously reported “spectral biomarkers” of lung cancer are Trp (1362 cm^−1^), Phe (1003 cm^−1^), protein (amide III, namely, for 785 nm laser line ~1217 cm^−1^ and 1273 cm^−1^ bands), and amide I bands (c.a. 1600–1683 cm^−1^), as well as collagen and phospholipid bands (1450 cm^−1^). Kowalska et al. reported that RS could differentiate lung cancers (patient n = 11) into (i) small versus non-small-cell carcinoma, and then a subset of NSCCs into (ii) squamous cell carcinoma versus adenocarcinoma versus large-cell carcinoma of the lung [29].

In our study, breast Ca presented a better RS signal with 785 nm than with the 532 nm laser line. The most characteristic signal was the Tyr doublet at 830 cm^−1^ and 858 cm^−1^, possibly caused by Tyr–heme interaction [14,15]. We also noted protein and biomatter signals (1200–1300 cm^−1^ and high-number regions, respectively; 785 nm laser line). In the literature, RS of breast carcinoma showed increased intensities of amide I (c.a. 1650 cm^−1^) and amide III (1220–1280 cm^−1^) bands, with reduced lipids (many bands), Tyr (1155 cm^−1^, 1517 cm^−1^), cysteine (538 cm^−1^), and nucleic acids (especially 676 cm^−1^—nucleic bases; 1080 cm^−1^—PO_2_) [30]. Breast cancer metastases to the lung were investigated by Bhattacharjee et al., who found that RS detected metastasis with 71% efficiency [31]. Breast cancer has various types and subtypes. The most important steps for treatment and prognosis are staging and division—according to estrogen, progesterone, and HER2 expression and proliferation count—into molecular subtypes of luminal A and B, HER-negative, and triple negative; these classifications have been investigated with RS, but data from more subjects are required [32].

We found increases in Phe, amide III, and lipid band intensities in OvCa, consistent with Frimpong et al.’s findings for tissue fragments. The cited study revealed an increase in Phe (1003 and 1205 cm^−1^) and nucleotides (1256, 1332, and 1573 cm^−1^) and a slight increase in lipids (545, 1074, 1119, and 1438 cm^−1^) and amide I groups (1652 cm^−1^) in cancer. Band intensities for proline and collagen (812, 856, 919, 935, and 1035 cm^−1^), Tyr (1159 cm^−1^), amide III groups (1240 and 1276 cm^−1^), CH_3_ modes in proteins (1400 and 1415 cm^−1^), and lipids and DNA (1465 cm^−1^) were decreased [33]. In pleural effusions from OvCa patients, collagen bands were not observed. Further, RS can predict ovarian cancer resistance to platinum therapy [34].

Colon adenocarcinoma pleural effusions produced bands of low intensity, except for the high-number biomatter region, some lipids (1450 cm^−1^ with 785 nm laser), and carbohydrates (1338 cm^−1^ with 532 nm laser). An in vivo colonoscopy RS investigation of colon AdCa with a 785 nm laser line showed increases in Phe (1003 and 1028 cm^−1^), Tyr (1162 cm^−1^; herein, for most collagen assignments, this finding might be explained by frequent tumor desmoplasia), and proteins and decreases in protein and lipid amide III (1262 cm^−1^) [35]. We found band shifts in effusion samples compared with in vivo experiments, which involved Trp and DNA (1333 cm^−1^ for in vivo), amide I (1657 cm^−1^), and lipids and phospholipids (1306 and 1442 cm^−1^).

Our RCC samples presented high Phe, 1450, and 1670 cm^−1^ band intensities. Raman spectra distinguished RCC from healthy renal and fat tissue with an accuracy of c.a. 93% [36]. Moreover, the aforementioned work reported the ability to differentiate RCC types and grades (accuracies of 88% and 90%, respectively). Both the cited work and our results show overall low signal intensity with the 785 nm laser line. We confirm that the difficulty is a similarly low signal for the 532 nm laser line as well. To solve this problem, dual IR and RS of patients’ serum has been investigated [37]. In the RCC group, IR showed decreased intensities of amide I (1650 cm^−1^) and amide II (1540 cm^−1^) bands; conversely, those of 1080 cm^−1^, 2931 cm^−1^, and 3286 cm^−1^ were increased.

Asbestosis minerals, such as crocidolite fibers, have also been the subject of RS [38,39]. We found infrared studies of mesothelioma, but no RS analyses. In their IR investigation of mesothelioma, Abbas et al. found higher levels of lipids (high-number bands), triglycerides and cholesterol (band 1739 cm^−1^), and proteins (amide I and amide II, but not amide III) compared to controls and other lung cancers [40].

### 3.4. Study Limitations

This study examined various malignant effusions, some of which are rare; e.g., mesothelioma incidence is 1:100,000 [41], and RCC constitutes 1–2% of malignant pleural effusions [42]. Due to these various occurrences, collecting a similar number of samples to represent different effusion types is difficult. A discriminant analysis for diagnostic applications should be performed on a larger subject group. The spectral variability for one patient in our study was low. Therefore, further discriminant analysis was not performed. We collected these samples over approximately one year and asked patients to undergo further multiple laboratory tests, radiology, and histopathological examinations. Usually, the best opportunity to collect fluid is at the beginning of the diagnostic process; as effusion recurs, it is treated with talc pleurodesis [43]. The tissue biopsies were taken after patient stabilization, under anesthesia, approximately 2 months after effusion collection and pleurodesis. We considered performing tissue measurements, but there was a problem with the small sample size (needed for immunohistochemistry) and necrosis, and we ultimately discontinued this line of study.

## 4. Materials and Methods

The study was approved by the First Local Ethical Committee at the Jagiellonian University Medical College in Krakow (No. 1072.6120.57.2023). Informed consent was obtained from each patient enrolled.

### 4.1. Experimental Samples

Effusion samples were collected from twenty adult patients with clinical suspicion of malignancy at the Thoratic Surgery Department. One part of each fluid sample remaining after decompression (volume 0.2–1.3 l) was sent fresh for standard diagnostic cytology (50 mL), and another part (100 mL) fixed with 99.9% ethanol (Kryptontek; with a ratio 1:1) was preserved for RS experiments. Of the spectroscopic sample, only 0.3 mL was dried and measured on CaF_2_ windows.

Grouping was based on hematoxylin–eosin cytology, medical history, and tests (reactive changes in controls), and malignancies were grouped according to histological examinations of tissue biopsies (with immunohistochemistry when needed).

### 4.2. Raman Spectroscopy

RS images were collected using a Confocal Raman Imaging WITec alpha 300M+, with a 20× long working distance objective (Zeiss, Epiplan Neofluar, NY, USA) and 600 and 300 gratings for 532 nm and 785 nm lasers, respectively. The laser power was set to 7 and 20 mW for the two lasers and tested to prevent sample degradation. The spectrometer was equipped with an air-cooled solid-state laser and a CCD detector, cooled to −60 °C. Each point was measured for 1 min (20 s × 5 averaging accumulations). Background subtraction was performed (400–1800 and 2800–3000 cm^−1^) with N order shape 150.

### 4.3. Data Analysis and Visualization

Raman data analysis was performed using the WITec software (WITec Plus, Ulm, Germany, Version 6.2). Data extracted from WITec text files were imported into Origin Pro (ver. 2024, OriginLab program, OriginLab Corporation, Northampton, MA, USA) with the PCA for Spectroscopy app (v1.44) software and then smoothed (Savitzky–Golay, 13 points, 2nd polynomial). PCA was performed on 60 spectra (20 patients × 3 spectra) collected with 532 nm and 785 nm laser lines in the 400–1720 cm^−1^ and 2800–3050 cm^−1^ regions, separately and together for the whole bioregion. The outcomes were presented using Origin.

## 5. Conclusions

RS holds great potential for the diagnosis of pleural effusion malignancies and examination of cells in patient-derived cultures for personalized medicine. Preparation and measurement are simple, as presented herein. Both laser lines captured the fingerprint region characteristic of cancer, optimal at c.a. 400–1720 cm^−1^. Fluorescence is low in this spectral region. Band intensity in high-number regions varies depending on whether the 532 nm or 785 nm laser line is used; 785 nm is likely preferable for samples with low organic matter and blood content. Further investigation is needed to explain the heme–Tyr interaction. All malignancy types investigated are biochemically characterized by increased amino acid residues. Small-cell carcinoma had the highest DNA content, and mesothelioma is characterized by high lipid content. Protein bands show discrepant content patterns for different chemical band vibration modes.

## Figures and Tables

**Table 1:** Detailed anonymized patient data from standard cytological findings.

Nr	Sex	Age [Years]	Side	Experimental Group	Color	MTC	SqC	Lym	Neu	MPh	NC
1	M	72	R	RCC	W	0	0	0	0	0	2
2	K	57	R	Mes	W	2	0	3	0	0	2
3	K	75	L	SqCa Lung	W	0	3	0	0	0	3
4	M	84	L	Control	W	0	0	1	3	2	0
5	K	61	R	Control	W	3	0	3	0	3	0
6	M	70	L	SqCa Lung	LB	1	3	0	0	0	3
7	M	73	L	Control	W	3	0	3	1	1	0
8	K	74	R	Control	W	2	0	2	0	1	0
9	M	67	L	Control	W	1	0	1	0	3	0
10	M	71	L	Mes	W	3	0	0	0	0	2
11	K	70	R	OvCa	LB	0	0	0	0	0	2
12	M	88	R	SCC Lung	B	0	0	1	1	0	2
13	K	67	R	Mes	W	0	0	0	0	0	3
14	K	64	R	OvCa	W	0	0	0	0	0	2
15	M	61	R	Mes	Y	3	0	0	0	0	3
16	M	70	R	AdCa Lung	LB	0	0	0	0	0	2
17	K	75	L	AdCa Lung	Y	1	0	3	2	2	3
18	M	65	L	AdCa Lung	W	0	0	0	0	0	1
19	M	80	R	AdCa Colon	W	2	0	1	1	2	1
20	K	75	R	Breast Ca	W	1	0	0	0	1	1

The scale for the presence of cells, proteins, and detritus ranges from 0 (absent) to 3 (large amount). Colors: W—white; B—brown; LB—light brown; Y—yellowish [fixed fresh blood is brown, lysed blood is yellow (due to heme oxidation), and protein-rich fluid is white (due to denaturation). Abbreviations: MTC—mesothelial cells (malignant and/or not malignant); SqC—squamous cells; Lym—lymphocytes; Neu—neutrophils; MPh—macrophages; NC—neoplasm cells.

**Table 2:** Biochemical components in various cancers and reactive changes (control) according to the spectra of the 532 nm laser line.

Effusion Cause	Biochemical Components and Bands Assigned to Them [cm^−1^]						
	Proteins	Nucleic Acids	Lipids	Carbohydrates	Trp	Phe	Tyr
	1232, 1362, 1395, 1450, 1583, 1670	458, 593, 750, 1362, 1553	667, 1450, 2875, 2934, 2974	667, 1338	750, 1126, 1316, 1362	1003, 1155	593, 830, 858, 946, 1155, 1517, 1628
Control	++	+	+	+	−/+	+	−/+
Mes	+	+	+++	+	−/+	+	+
AdCa Lung	+	+	−/+	−	−/+	++	+++
AdCa Colon	−/+	+	−/+	+	−/+	++	+
SqCa Lung	+++	++	++	+++	++	+++	++
OvCa	++	+	+++	+	+	++	−
Breast Ca	+	+	−/+	−	−/+	−/+	+
RCC	++	+	++	+	−/+	++	−
SCC Lung	+++	+++	−/+	++	+++	+	++

Number of pluses presents biocomponents levels deciphered from Raman band intensity (assignments mainly according to Pezzotti G. [15] and Ishimaru et al. [16]). Pluses (+) denote high intensity, and minuses (−/+) denote low intensity or the absence of a band (−).

## Data Availability

Dataset available on request from the authors.

## Abbreviations

The following abbreviations are used in this manuscript:

RS	Raman Spectroscopy
Mes	Mesothelioma
AdCa	Adenocarcinoma (of the lung or colon)
SqCa	Squamous Carcinoma
OvCa	Ovarian Carcinoma (serous type)
SCC	Small-Cell Carcinoma
RCC	Renal Cell Carcinoma
PCA	Principal Component Analysis

## References

[1] Korczyński P., Górska K., Konopka D., Al-Haj D., Filipiak K.J., Krenke R. (2020). Significance of congestive heart failure as a cause of pleural effusion: Pilot data from a large multidisciplinary teaching hospital. Cardiol. J..

[2] Sears D., Hajdu S.I. (1987). The cytologic diagnosis of malignant neoplasms in pleural and peritoneal effusions. Acta Cytol..

[3] Monte S.A., Ehya H., Lang W.R. (1987). Positive effusion cytology as the initial presentation of malignancy. Acta Cytol..

[4] Bray F., Laversanne M., Sung H., Ferlay J., Siegel R.L., Soerjomataram I., Jemal A. (2024). Global cancer statistics 2022: GLOBOCAN estimates of incidence and mortality worldwide for 36 cancers in 185 countries. CA Cancer J. Clin..

[5] Bibby A.C., Tsim S., Kanellakis N., Ball H., Talbot D.C., Blyth K.G., Maskell N.A., Psallidas I. (2016). Malignant pleural mesothelioma: An update on investigation, diagnosis and treatment. Eur. Respir. Rev..

[6] Amin M.B., Edge S., Greene F., Byrd D.R., Brookland R.K., Washington M.K., Gershenwald J.E., Compton C.C., Hess K.R., Sullivan D.C. (2017). AJCC Cancer Staging Manual.

[7] Kumar M.C., Ghosh M., Chowdhury J. (2026). Fabrication of gold nano-particles embedded in the Langmuir Blodgett film of Poly (methyl methacrylate) for rapid screening of melamine adulterant in milk powder. Opt. Mater..

[8] Hardy M., Chu H.O.M. (2025). Laser wavelength selection in Raman spectroscopy. Analyst.

[9] Giamougiannis P., Morais C.L., Grabowska R., Ashton K.M., Wood N.J., Martin-Hirsch P.L., Martin F.L. (2021). A comparative analysis of different biofluids towards ovarian cancer diagnosis using Raman microspectroscopy. Anal. Bioanal. Chem..

[10] Kujdowicz M., Placha W., Mech B., Chrabaszcz K., Okoń K., Malek K. (2021). In Vitro Spectroscopy-Based Profiling of Urothelial Carcinoma: A Fourier Transform Infrared and Raman Imaging Study. Cancers.

[11] Qi Y., Liu Y., Luo J. (2023). Recent application of Raman spectroscopy in tumor diagnosis: From conventional methods to artificial intelligence fusion. PhotoniX.

[12] Hauptmann A., Hoelzl G., Mueller M., Bechtold-Peters K., Loerting T. (2023). Raman Marker Bands for Secondary Structure Changes of Frozen Therapeutic Monoclonal Antibody Formulations During Thawing. J. Pharm. Sci..

[13] Sjöberg B., Foley S., Cardey B., Enescu M. (2014). An experimental and theoretical study of the amino acid side chain Raman bands in proteins. Spectrochim. Acta-Part A Mol. Biomol. Spectrosc..

[14] Hernández B., Coïc Y.M., Kruglik S.G., Sanchez-Cortes S., Ghomi M. (2024). The relationship between the tyrosine residue 850–830 cm^−1^ Raman doublet intensity ratio and the aromatic side chain χ1 torsion angle. Spectrochim. Acta Part A Mol. Biomol. Spectrosc..

[15] Pezzotti G. (2021). Raman spectroscopy in cell biology and microbiology. J. Raman Spectrosc..

[16] Ishimaru Y., Oshima Y., Imai Y., Iimura T., Takanezawa S., Hino K., Miura H. (2018). Raman Spectroscopic Analysis to Detect Reduced Bone Quality after Sciatic Neurectomy in Mice. Molecules.

[17] Brauchle E., Schenke-Layland K. (2018). Raman spectroscopy in biomedicine—Non-invasive in vitro analysis of cells and extracellular matrix components in tissues. Biotechnol. J..

[18] Kujdowicz M., Perez-Guaita D., Chlosta P., Okon K., Malek K. (2023). Evaluation of grade and invasiveness of bladder urothelial carcinoma using infrared imaging and machine learning. Analyst.

[19] Kowalska A.A., Czaplicka M., Nowicka A.B., Chmielewska I., Kędra K., Szymborski T., Kamińska A. (2022). Lung Cancer: Spectral and Numerical Differentiation among Benign and Malignant Pleural Effusions Based on the Surface-Enhanced Raman Spectroscopy. Biomedicines.

[20] Liu K., Jin S., Song Z., Jiang L. (2020). High accuracy detection of malignant pleural effusion based on label-free surface-enhanced Raman spectroscopy and multivariate statistical analysis. Spectrochim. Acta-Part A Mol. Biomol. Spectrosc..

[21] Shamseddin M., Obacz J., Garnett M.J., Rintoul R.C., Francies H.E., Marciniak S.J. (2021). Use of preclinical models for malignant pleural mesothelioma. Thorax.

[22] Zipprick J., Demir E., Krynska H., Köprülüoğlu S., Strauß K., Skribek M., Hutyra-gram Ötvös R., Gad A.K., Dobra K. (2025). Ex-Vivo Drug-Sensitivity Testing to Predict Clinical Response in Non-Small Cell Lung Cancer and Pleural Mesothelioma: A Systematic Review and Narrative Synthesis. Cancers.

[23] Shatar L., Pillai N., Chee H.Y., Mustafa F.H., Nasir M.N., Zakaria R., Mustafa M.K., Zain M.N., Suhailin F.H. (2025). Enhancement in surface-enhanced Raman spectroscopy (SERS) substrates and the potential for diseases detection. Appl. Spectrosc. Rev..

[24] Pimenta S., Correia J.H. (2025). Biomedical Applications of Raman Spectroscopy: A Review. Photochem.

[25] Cialla-May D., Bonifacio A., Markin A., Markina N., Fornasaro S., Dwivedi A., Dib T., Farnesi E., Liu C., Ghosh A. (2024). Recent advances of surface enhanced Raman spectroscopy (SERS) in optical biosensing. TrAC Trends Anal. Chem..

[26] Lu D., Huang Z., Chen J., Lu Y. (2025). pH-Adjusted Liquid SERS Approach: Toward a Reliable Plasma-Based Early Stage Lung Cancer Detection. Anal. Chem..

[27] Sharma N., Rao S., Noothalapati H., Mazumder N., Paul B. (2025). Raman spectroscopy in the detection and diagnosis of lung cancer: A meta-analysis. Lasers Med. Sci..

[28] Chen C., Hao J., Hao X., Xu W., Xiao C., Zhang J., Pu Q., Liu L. (2021). The accuracy of Raman spectroscopy in the diagnosis of lung cancer: A systematic review and meta-analysis. Transl. Cancer Res..

[29] Kowalska A.A., Czaplicka M., Chmielewska I., Pankowski J., Kaminska A. (2025). The Lung Cancer Detection and Type Determination From Plasma and Lung Tissues by Surface-Enhanced Raman Spectroscopy. J. Raman Spectrosc..

[30] Hanna K., Krzoska E., Shaaban A.M., Muirhead D., Abu-Eid R., Speirs V. (2022). Raman spectroscopy: Current applications in breast cancer diagnosis, challenges and future prospects. Br. J. Cancer.

[31] Bhattacharjee T., Tawde S., Hudlikar R., Mahimkar M., Maru G., Ingle A., Murali Krishna C. (2015). Ex vivo Raman spectroscopic study of breast metastatic lesions in lungs in animal models. J. Biomed. Opt..

[32] Melitto A.S., Arias V.E.A., Shida J.Y., Gebrim L.H., Silveira L. (2022). Diagnosing molecular subtypes of breast cancer by means of Raman spectroscopy. Lasers Surg. Med..

[33] Frimpong D., Shore A.C., Gardner B., Newton C., Pawade J., Frost J., Atherton L., Stone N. (2025). Raman spectroscopy of ovarian and peritoneal tissue in the assessment of ovarian cancer. Analyst.

[34] Kluz-Barłowska M., Kluz T., Paja W., Pancerz K., Łączyńska-Madera M., Miziak P., Cebulski J., Depciuch J. (2014). FT-Raman and FTIR spectroscopy as a tools showing marker of platinum-resistant phenomena in women suffering from ovarian cancer. Sci. Rep..

[35] Fousková M., Vališ J., Synytsya A., Habartová L., Petrtýl J., Petruželka L., Setnička V. (2023). In vivo Raman spectroscopy in the diagnostics of colon cancer. Analyst.

[36] He C., Wu X., Zhou J., Chen Y., Ye J. (2021). Raman optical identification of renal cell carcinoma via machine learning. Spectrochim. Acta-Part A Mol. Biomol. Spectrosc..

[37] Chen C., Chen F., Yang B., Zhang K., Lv X., Chen C. (2022). A novel diagnostic method: FT-IR, Raman and derivative spectroscopy fusion technology for the rapid diagnosis of renal cell carcinoma serum. Spectrochim. Acta Part A Mol. Biomol. Spectrosc..

[38] Musa M., Croce A., Allegrina M., Rinaudo C., Belluso E., Bellis D., Toffalorio F., Veronesi G. (2012). The use of Raman spectroscopy to identify inorganic phases in iatrogenic pathological lesions of patients with malignant pleural mesothelioma. Vib. Spectrosc..

[39] Bard D., Yarwood J., Tylee B. (1997). Asbestos fibre identification by Raman microspectroscopy. J. Raman Spectrosc..

[40] Abbas S., Simsek Ozek N., Emri S., Koksal D., Severcan M., Severcan F. (2018). Diagnosis of malignant pleural mesothelioma from pleural fluid by Fourier transform-infrared spectroscopy coupled with chemometrics. J. Biomed. Opt..

[41] Huang J., Chan S.C., Pang W.S., Chow S.H., Lok V., Zhang L., Lin X., Lucero-Prisno D.E., Xu W., Zheng Z.J. (2023). Global Incidence, Risk Factors, and Temporal Trends of Mesothelioma: A Population-Based Study. J. Thorac. Oncol..

[42] Wasifuddin M., Ilerhunmwuwa N., Hakobyan N., Sedeta E., Uche I., Aiwuyo H.O., Perry J.C., Heravi O., Boris A. (2023). Malignant Pleural Effusion As the Initial Presentation of Renal Cell Carcinoma: A Case Report and Literature Review. Cureus.

[43] Xia H., Wang X.J., Zhou Q., Shi H.Z., Tong Z.H. (2014). Efficacy and safety of talc pleurodesis for malignant pleural effusion: A meta-analysis. PLoS ONE.

